# Detection of *Trypanosoma cruzi* by PCR in adults with chronic Chagas disease treated with nifurtimox

**DOI:** 10.1371/journal.pone.0221100

**Published:** 2019-08-21

**Authors:** Camilo Vergara, Gabriela Muñoz, Gabriela Martínez, Werner Apt, Inés Zulantay

**Affiliations:** Laboratorio de Parasitología Básico-Clínico, Programa de Biología Celular y Molecular, Instituto de Ciencias Biomédicas, Facultad de Medicina, Universidad de Chile, Santiago, Chile; Philipps-Universitat Marburg, GERMANY

## Abstract

Chagas disease, a vector-borne parasitosis caused by *Trypanosoma cruzi*, is endemic to Latin America and has spread to other countries due to immigration of infected persons. It is estimated that 160,000 people are infected in Chile, most of them in the chronic phase and without etiological treatment. The infection is confirmed by conventional serological methods while molecular methods have become in valuable tools to evaluate parasitemia in treated and non-treated chronic Chagas disease patients. The objective of this study was to determine, by conventional Polymerase Chain Reaction, the presence of *T*. *cruzi* kinetoplastid DNA in peripheral blood samples from 114 adult individuals with confirmed chronic Chagas disease, before and 6.6 years (average) after treatment with nifurtimox. The samples were received and preserved in guanidine-EDTA until DNA purification. Conventional PCR assays were performed in triplicate with *T*. *cruzi* kinetoplastid DNA primers 121 and 122. The amplified products were fractionated by electrophoresis in 2% agarose gels. A 330 bp product represented a positive assay. 84.2% (96 cases) and 6.1% (7 cases) of the samples taken before and after the treatment, respectively, were positive. The McNemar test showed a statistically significant difference between the groups of samples (p<0.001). Since serological negativization (the current cure criterion) delay many years after therapy and positive parasitological results represent a treatment failure, the conversion of pre-therapy positive conventional PCR is a qualitative and complementary tool that could be included in protocols of prolonged follow-up.

## Introduction

Chagas disease (ChD), caused by *Trypanosoma cruzi*, remains a major public health issue in endemic areas where international collaborative efforts initiatives have been carried out to improve socioeconomic and household conditions, blood banks screenings and vector control [1,2]. Currently, it is estimated that Chagas disease affects between 6 and 8 million individuals, with an estimated number of deaths of approximately 12,000 per year worldwide [3]. The pathogenesis of ChD is not completely understood, but seems to depend on a complex interaction between host and parasite [4]. Pathogenic mechanisms that develop clinical signs in patients in the chronic phase is still an issue in debate, nonetheless, persistence of the parasite seems to be responsible of the progress of the damage [4–8].

In the chronic phase of the disease, when parasitemia falls, the diagnosis is mainly performed by conventional serological methods and is based on the positivity of, at least two different tests [9]. The parasitological methods xenodiagnosis and hemoculture, of low sensitivity in this infection phase, have been replaced by molecular methods, as conventional Polymerase Chain Reaction (cPCR) and real time PCR, both have proved to be useful parasitological tools for evaluation of trypanocide efficacy [9–12].

It is estimated that parasitological and serological cure with nifurtimox (NF) or benznidazole (BZ) is reached in up to 98–100% of infected neonates and in 70–75% of the patients treated in the acute phase [13], while the percentages of serological and/or parasitological reversion in the chronic phase are not conclusive [14,15].

This study aims to compare the parasitological condition of patients, before and after (6.6 years on average) treatment with NF using qualitative detection of *T*. *cruzi* DNA by cPCR assays.

## Materials and methods

### Studied population

114 adult individuals between the ages of 18 and 70 years (average 43.7) with chronic ChD confirmed by ELISA and indirect immunofluorescence were selected according to the inclusion and exclusion criteria of the study protocol. All the patients lived in urban and rural areas of the Provinces Choapa and Limarí, Coquimbo Region, Chile, an endemic area located between 29° 02’ and 32° 16’ South latitude. Each patient was treated between 2009 and 2012 with NF at a dose of 8 mg/kg/day in two daily intakes during 60 days, in agreement with international consensus [16].

### Ethics statement

Every participant in this study signed two written informed consents, before the treatment (Resolution 046/2009 Project Fondecyt 1100768) and previous to the prolonged follow-up (Resolution 012/16 Project Fondecyt 1161485). Both were approved by the Ethics Committee of the Faculty of Medicine of the University of Chile.

### Biological samples

Peripheral blood samples were collected before starting the treatment and in January 2017, 5.2 to 8.0 years after the treatment (average 6.6 years), in rural or urban health centres (S1 Table). A five mL blood sample was mixed and preserved with an equal volume of 6 M guanidine-HCl 0.2 M EDTA solution, incubated at 98°C for 15 min. and stored at 4°C until DNA extraction 4–5 days later.

### DNA extraction

DNA extraction was performed with an initial volume of 200 μL of the sample mixture using the High Pure PCR Template Preparation Kit (Roche Diagnostics, USA) according to the manufacturer’s instructions. The purified DNA obtained was maintained at -80°C. Work aliquots were stored at -20°C.

### Conventional PCR assays

cPCR was performed as previously described [17], using oligonucleotides 121 (5’-AAATAATGTACGGGKGAGATGCATGA-3’) and 122 (5’-GGTTCGATTGGGGTTGGGTAATATA-3’), to amplify the variable regions of minicircle kinetoplast DNA (kDNA) of *T*. *cruzi* [18]. Positivity criterion was the presence of a 330 bp specific band for *T*. *cruzi* kDNA in at least two of three assays to obtain 330 bp amplicons.

### Statistical analysis

The McNemar test with a significance level of 0.05 was applied to compare proportions of positive and negative samples determined by cPCR, before and after treatment with NF.

## Results

**Fig 1:**
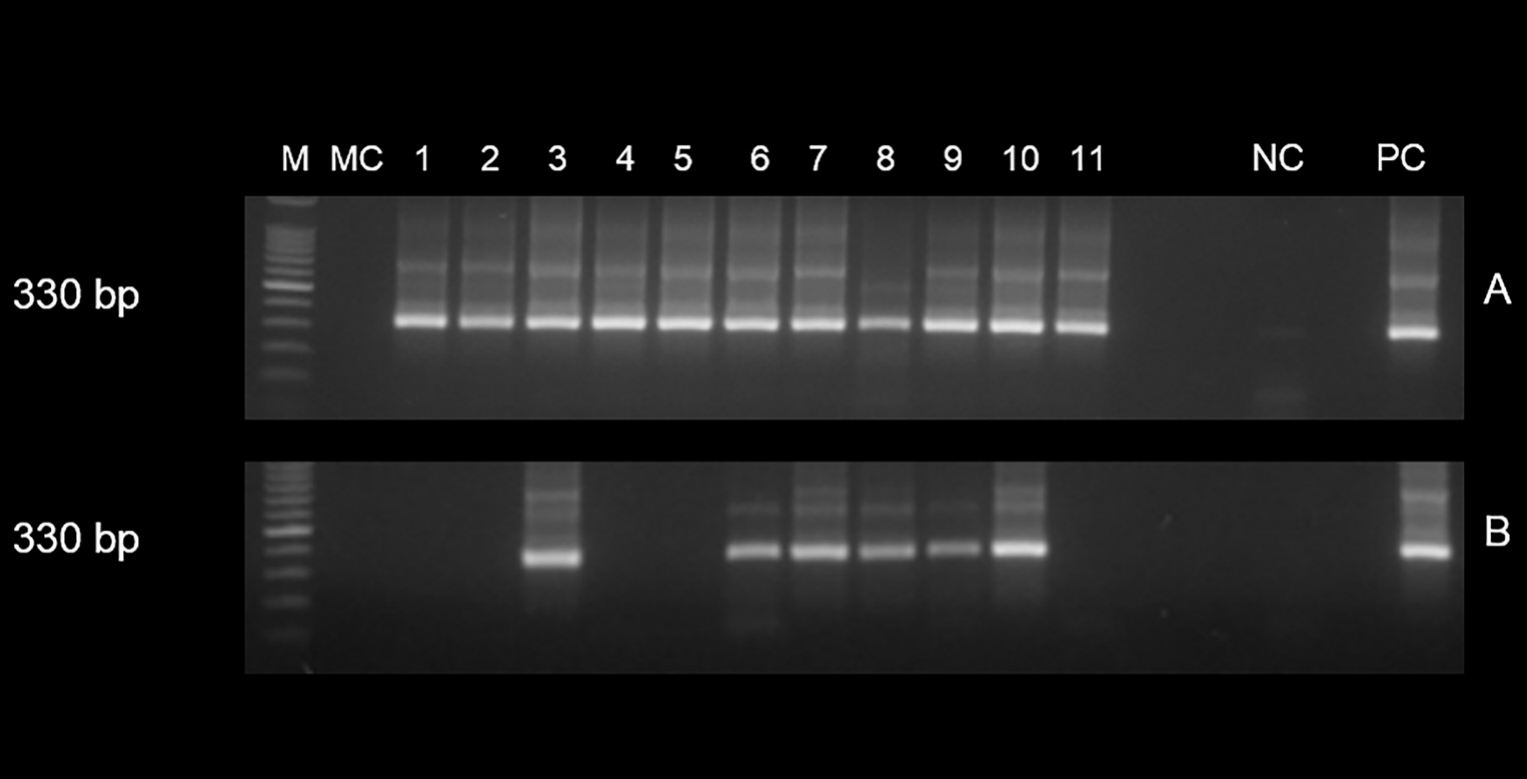
Conventional PCR with 121–122 primers to amplify variable regions of minicircle of *Trypanosoma cruzi* kDNA in 11 chilean patients with chronic Chagas disease treated with nifurtimox.

*T*. *cruzi* kDNA was detected in 96 of the 114 (84.2%) pre-treatment cases, while in the post-treatment, only 7 individuals (6.1%) showed the 330 bp specific band (Fig 1). Seventeen patients (14.9%) presented a negative cPCR before being treated, and this status was maintained in the post-treatment. Six (5.3%) individuals maintained the positive condition despite the treatment. Finally, one case (0.9%) that had a negative cPCR assay before the treatment, was positive after treatment (not shown in Fig 1). The proportion of positive cPCR cases after the NF treatment showed a statistically significant difference compared to positive cases before treatment (p<0.0001) (Table 1).

**Table 1:** Treatment of chronic Chagas disease with nifurtimox. Conventional PCR results before and after treatment in prolonged follow-up (6.6 years average).

		After treatment		
		Positive	Negative	Total
	**Positive**	6 (5.3%)	90 (78.9%)	96 (84.2%)
**Before treatment**				
	**Negative**	1 (0.9%)	17 (14.9%)	18 (15.8%)
	**Total**	7 (6.1%)	107 (93.9%)	114 (100%)

(p<0.0001)

Electrophoretic pattern of minicircle region 330 bp in size of *T*. *cruzi* kDNA-cPCR amplified with primers 121 and 122 from 11 Chilean patients with chronic Chagas disease treated with nifurtimox evaluated in prolonged follow-up. (A) Positive cPCR of 11 cases in pre-therapy condition (B) 6 cases with positive cPCR before and after therapy, and 5 cases with positive and negative cPCR before and after treatment, respectively. M: 100 base pair ladder; MC: PCR mix control; NC: negative control (DNA of individual without Chagas disease); PC: positive control (DNA *T*. *cruzi* Tulahuen strain).

## Discussion

The results obtained in this study show that 90 out of 96 cases (93.8%) with positive cPCR in pre-therapy have negative cPCR in the 6.6 years average follow-up control. This is concordant with others studies. In Switzerland, thirty-seven Latin American migrants with chronic ChD were treated with NF and followed-up three years later. All patients had negative cPCR results but one (2.7%) was positive with real-time PCR [19]. A study conducted in Brazil on individuals with chronic ChD treated with NF or BZ demonstrated that 31 out of 48 patients (64.6%) had negative cPCR results in an average of 20 years of follow-up [20]. On the other hand, of 29 people with chronic ChD who completed successfully a treatment with BZ, 48.3% showed no amplification of *T*. cruzi DNA by nested PCR in the follow-up, while 10.3% of the studied samples were considered inconclusive [21]. Murcia *et al*. demonstrated a negative conversion of cPCR in 96 Bolivians migrants with chronic ChD, 90 days after treatment with BZ [22]. The same author, shows that 96.7% of Bolivian migrants with chronic ChD followed 7 years post-therapy with BZ, maintained a negative cPCR [23]. Finally, Cardoso *et al*., described that 83.4% of individuals with chronic ChD treated with BZ had negative results by real-time PCR after two years follow-up [24]. Although there are differences in the drug administration protocols, age of the patients and parasitological methods of *T*. *cruzi* detection in the mentioned studies conducted in the last two decades, there is concordance in high percentages of parasitological negativization in patients with chronic ChD treated with NF or BZ.

Regarding the case with negative and positive cPCR results before and after treatment, respectively, there are other similar cases in the literature [22]. cPCR success depends on the burden of circulating parasites in a patient’s bloodstream. As *T*. *cruzi* circulates in very small amounts in the chronic phase, a false negative cannot be discarded. In the present study, 84.2% positive cPCR in pre-therapy conditions is concordant with the sensitivity described for the method [25–27]. No related infections were included (Leishmaniasis or *T*. *rangeli*), since they are absent in Chile [25,28]. The 121 and 122 primers cannot amplify the target in *T*. *rangeli* efficiently due to mismatches in the 3' end of the primer, therefore the possibility of *T*. *rangeli* mis-amplification is remote [26]. Finally, there are exceptional reports of dwellings in Chile with intrusion (but not colonization) of infected vectors, in spite of the interruption of the domestic cycle in 1999 [29].

A single point of study in a post-therapy follow-up is not sufficient to conclude that the parasite is absent, for this reason it is necessary to investigate the parasitological evolution at several times in the follow-up, applying qualitative and quantitative parasitological methods in order to confirm positive or negative laboratory findings.

## Conclusions

There was a statistically significant difference in the proportion of positive cases by cPCR before and after therapy with NF in the studied population.

Although *T*. *cruzi* was not detected in 93.9% of the cases after treatment, all of them maintained positive serology [30], for this reason, it is not possible to interpret the obtained results as parasitological cure according to the current cure criterion.

The results of the present study demonstrated therapy failure in 6.1% of the cases.

Some authors recommend cPCR for chronic Chagas disease diagnosis only when serological tests are inconclusive [26], but we consider that qualitative detection of circulating *T*. *cruzi* could be a complementary tool to quantitative methods such as real-time PCR, which is more expensive and not easily accessible.

## Supporting information

S1 Table Cases data. Case, sex, city and origin area, conventional PCR results, age at treatment and follow-up period of 114 Chilean individuals with chronic Chagas disease treated with nifurtimox.
(XLSX)
